# Supplementary material for: Highly Sensitive and Ultra-Responsive Humidity Sensors Based on Graphene Oxide Active Layers and High Surface Area Laser-Induced Graphene Electrodes
Source: Nanomaterials (Basel). 2022 Aug 4;12(15):2684. doi: 10.3390/nano12152684 (PMC9370464; doi:10.3390/nano12152684)
Supplement: Supplementary file 1 [file nanomaterials-12-02684-s001.zip › nanomaterials-1802367-supplementary.pdf]

Supporting Information

Highly Sensitive and Ultra-Responsive Humidity Sensors Based on Graphene Oxide Active Layers and High Surface Area Laser Induced Graphene Electrodes

George Paterakis,[a,c] Eoghan Vaughan,[b] Dinesh R. Gawade,[b] Richard Murry,[b] George Gorgolis,[a] Stefanos Matsalis,[a,c] George Anagnostopoulos,[a] John L. Buckley,[b] Brendan O'Flynn,[b] Aidan J. Quinn,[b] Daniela Iacopino,[b]* Costas Galiotis[a,c]

[a]Institute of Chemical Engineering Sciences, Foundation for Research and Technology-Hellas (FORTH/ICE-HT), Patras 265 04, Greece

[b]Tyndall National Institute, University College Cork, Dyke Parade, Cork, Ireland

[c]Department of Chemical Engineering, University of Patras, Patras 26504, Greece

Details of LIG electrode geometries and sizes

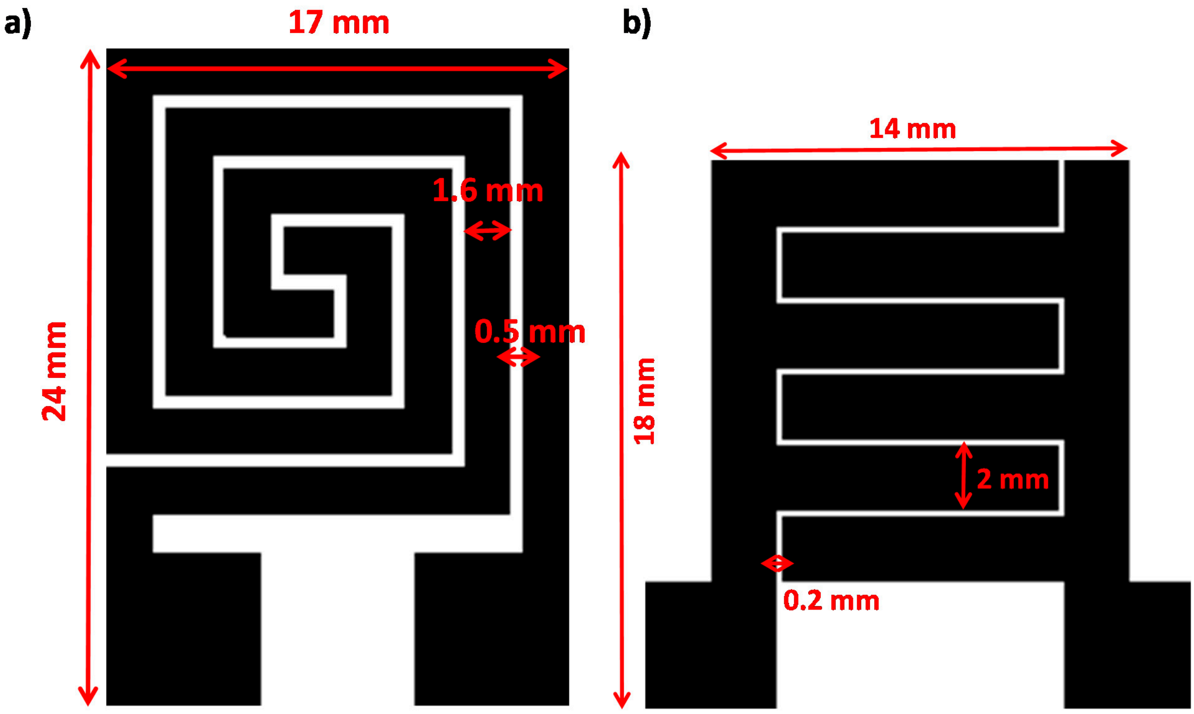

Figure S1. Schematic of a) spiral and b) interdigitated LIG electrode geometries and sizes used in this work.

Graphene Oxide synthesis and characterization

GO synthesis was performed in tandem with quality control, where spectroscopic and morphological characterization techniques such as XPS, Raman, SEM and AFM were used, to achieve the desired quality. The XPS analysis of the C1s peak spectra of GO (Figure S2 (a)) shows evidence for the presence of hydroxyls (-OH) and epoxides (C-O-C), usually appeared in the main structure of the matrix, as well as carboxyl groups (- COOH) detected at the edges [1]. The relative atomic concentration O/C was calculated as 0.6 by using the area ratio of the peak O 1s and C 1s. In addition, Figure S2 (b) shows a characteristic Raman spectrum of GO, which comprises several frequency bands, each one assigned to a certain structural configuration [2]. The main Raman peaks are the so-called G peak located at 1580 cm^{-1} and assigned to the shear in-plane mode of the graphite crystal, the D peak appearing at about 1360 cm^{-1}, attributed to the presence of structural defects of graphene lattice [3], and the 2D peak at 2680 cm^{-1}, which is the second-order overtone of the D peak. The intensity ratio of the D and G peaks was found to be equal to 0.97 ± 0.01 and can be correlated with the sp^3/sp^2 ratio on the GO lattice [4]. Furthermore, for the analysis of the morphological characteristics and the lateral size of the flakes, GO was characterized both by SEM (Figure S2 (c)) and AFM (Figure S2 (d)). From the analysis, it was found that the thickness of the flakes was appeared at ~1.2 nm, while the lateral size was below 2 μm and in most of the cases in the range of a few hundreds of nm.

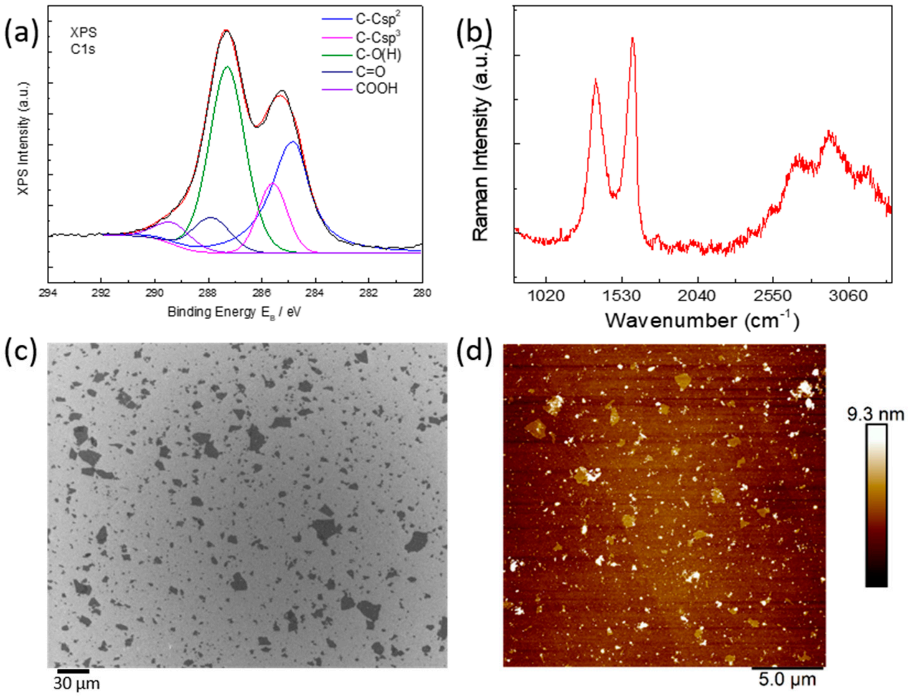

Figure S2 Spectroscopic and morphological characterization of GO by (*a*) XPS, (*b*) Raman, (*c*) SEM and (*d*) AFM.

Spiral LIG/GO humidity sensor stability measurements

Figure S3 shows the capacitance vs time stability measurements performed on four different class of sensors fabricated by drop casting 150 µl of 0.33 mg/ml, 0.67 mg/ml, 1.33 mg/ml and 2 mg/ml GO dispersions on spiral LIG electrodes. For each GO concentration, stability measurements were carried out at 25, 50 and 75% RH over 30 min periods.

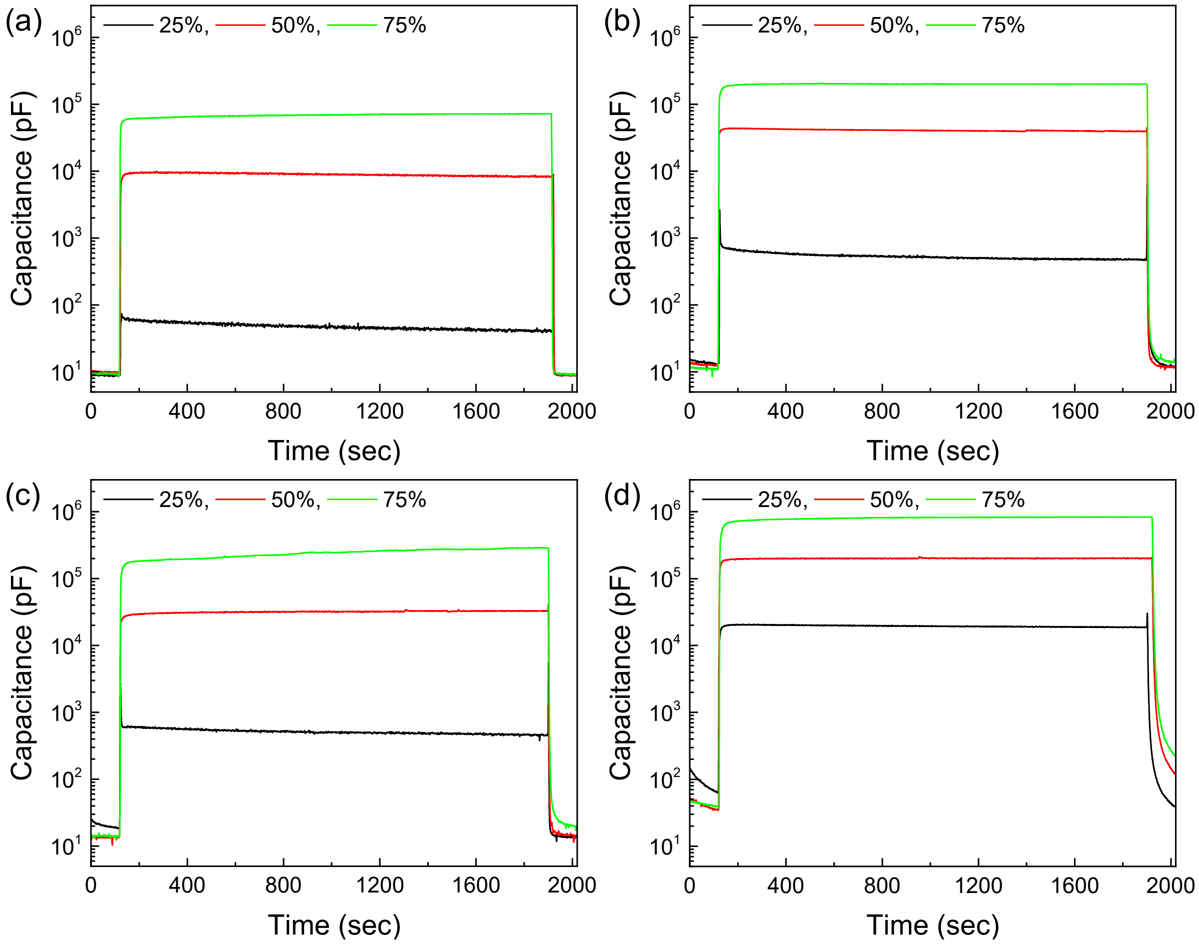

Figure S3. Stability measurements at 25, 50 and 75% RH performed on LIG/GO sensors containing (a) 0.33 mg/ml, (b) 0.67 mg/ml, (c) 1.33 mg/ml and (d) 2 mg/ml GO dispersions.

GO films characterization

Figure S4 shows thickness of GO films deposited on LIG spiral electrodes, measured by AFM and ranging from ca. 60 nm for the lowest GO concentration (0.33 mg/ml) to 1.7 µm for the highest GO concentration (2 mg/ml).

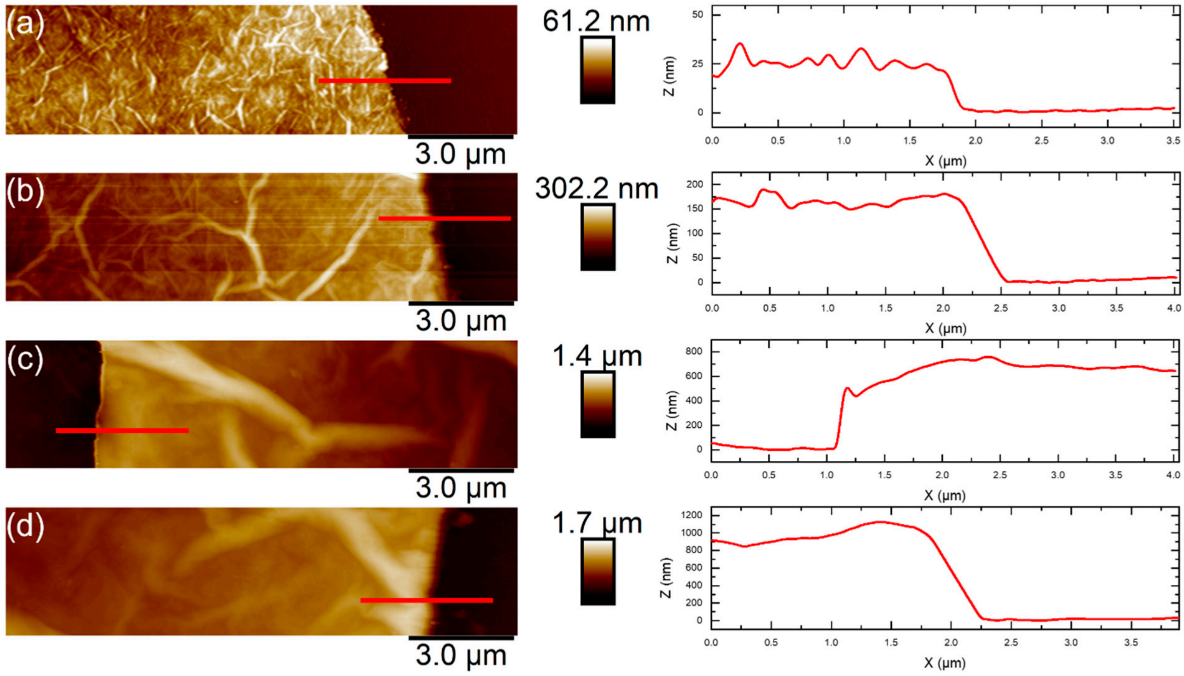

Figure S4. AFM thickness measurements of different GO sensing films obtained by drop casting of 150 µl GO dispersions of different concentrations: (a) 0.33 mg/ml, (b) 0.67 mg/ml, (c) 1.33 mg/ml, and (d) 2 mg/ml..

Spiral LIG/GO humidity sensors Capacitance vs RH behaviour

Figure S5 shows different capacitance vs RH responses obtained for LIG/GO sensors fabricated by drop deposition of 150 µl GO dispersions of various concentrations over LIG spiral electrodes. The range of investigated frequencies was 20 -100,000 Hz.

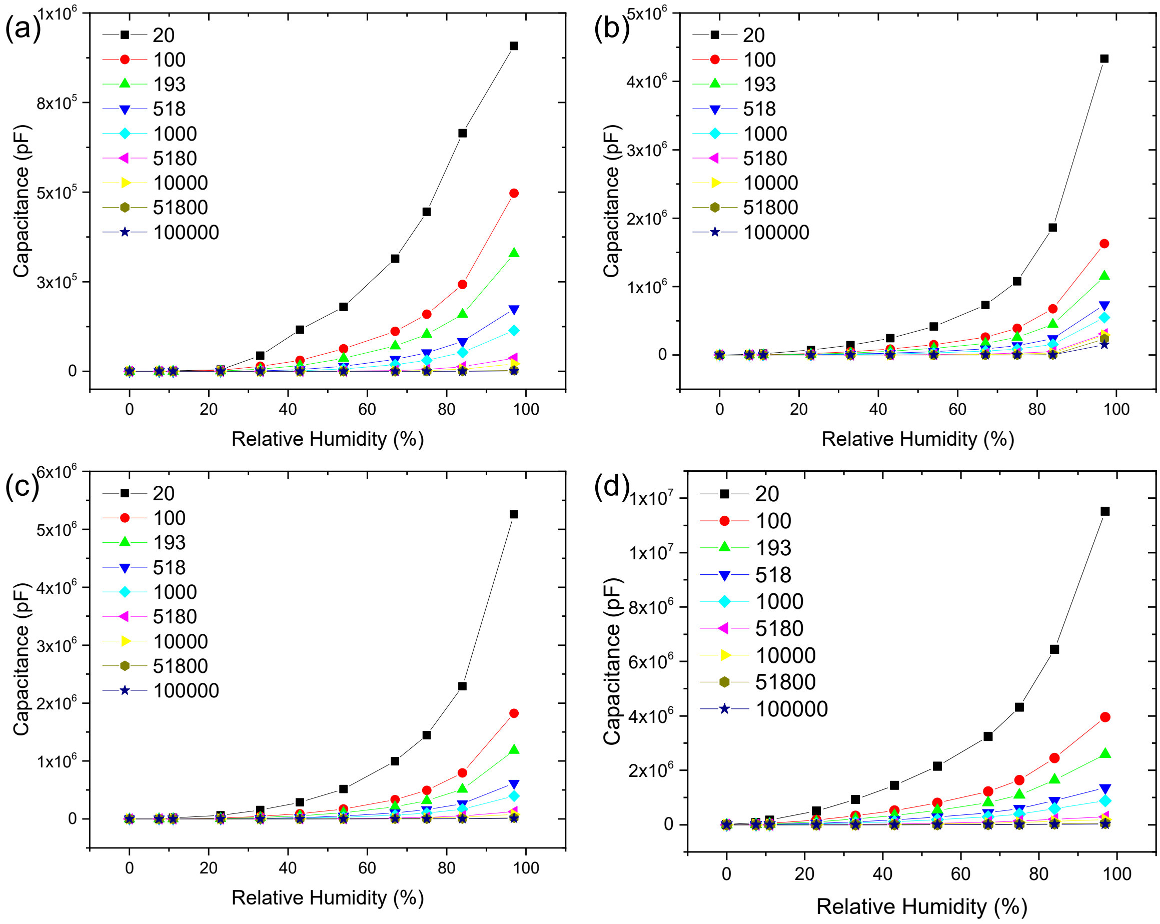

Figure S5. Capacitance vs RH at different frequencies for the LIG/GO sensors containing (a) 0.33 mg/ml, (b) 0.67 mg/ml, (c) 1.33 mg/ml and (d) 2 mg/ml GO active layers.

Table S1. Capacitance at 97% RH, Normalized Response and Sensitivity at the range of 0-97% RH, , measured at 500 Hz.

[mg/ml]	Capacitance @ 97% RH	N. Response (0 - 97%)	Sensitivity (0 - 97%)
	(pF)	(%) x 10^3	(pF/%RH)
0.33	175×10^3	1824	1800
0.67	346×10^3	3430	3560
1.33	521×10^3	3604	5370
2	1305×10^3	11710	13460

Table S2. Response/recovery times for all fabricated sensors in the range 20 – 80% RH

	Response time (s)				Recovery time (s)			
**Relative humidity (%)**	**20**	**40**	**60**	**80**	**20**	**40**	**60**	**80**
Spiral $GO_{0.33\ mg/ml}$	1	6	14	16	5	6	9	9
Spiral $GO_{0.67\ mg/ml}$	2	6	14	41	17	28	40	34
Spiral $GO_{1.33\ mg/ml}$	3	8	19	33	10	18	32	34
Spiral $GO_{2.0\ mg/ml}$	20	14	22	43	96	99	102	109
Interdigitated $GO_{0.33\ mg/ml}$	4	22	44	51	5	9	17	18

Table S3. Normalized sensitivity calculated for all fabricated sensors in the 20 – 1000 Hz frequency range.

	Sensitivity (pF/% RH)					
**Resonant frequency (Hz)**	**20**	**50**	**100**	**193**	**518**	**1000**
Spiral $GO_{0.33\ mg/ml}$	9361	7709	5122	3385	1802	1177
Spiral $GO_{0.67\ mg/ml}$	30586	23596	11306	8213	4538	2361
Spiral $GO_{1.33\ mg/ml}$	54232	28884	18794	12208	6354	4077
Spiral $GO_{2.0\ mg/ml}$	118726	62494	40780	26657	14024	9093
Interdigitated $GO_{0.33\ mg/ml}$	17152	9668	6538	4401	2365	1528

It should be noted that from the values of Table S3 all the sensors showed higher sensitivity at 518 Hz compared to the values in Table S1 at 500 Hz. This happened because the values in Table S3 were extracted from the measurements of Figure 5, where each sensor remained for 10 min at each RH level. On the other hand, the values in Table S1 came from the dynamic response measurements (Fig 2c, 4a-c & 5a), where all the sensors remained 90s at each RH level. Therefore, as these sensors showed a high response time in the gas flow system at 80%, they will show an even higher response at 97 % using the saturated solutions, resulting in a larger capacity.

Interdigitated LIG/GO humidity sensors performance

Figure S6 shows the performance characteristics of LIG/GO sensors obtained by drop casting 150 µl of GO 0.33 mg/ml on LIG interdigitated electrodes. Specifically, Figure S6,a shows the capacitance vs RH dependence measured between 20-100,000 Hz, Figure S6,b shows the impedance plots obtained at various RH values and Figure S6c shows repetitive absorption/desorption curves recorded in the 20 – 80 % RH interval.

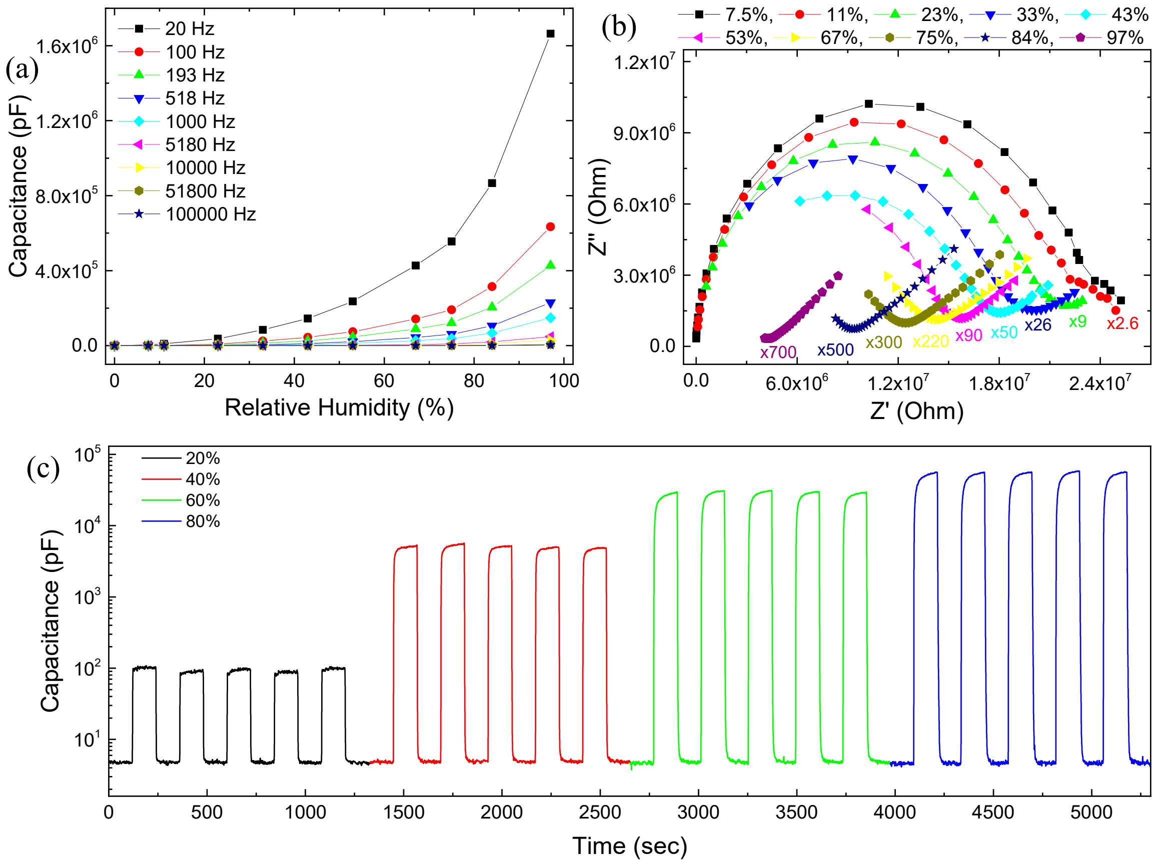

Figure S6 Performance characteristics of the LIG/GO interdigitated electrodes humidity sensor (a) Capacitance vs relative humidity for various frequencies, (b) Complex impedance plots at varied RH levels and (c) Repetitive absorption and desorption curves for 0 - 20 %, 0 - 40 %, 0 - 60 % and 0 - 80 % RH. All the measurements were performed at 500 mV.

Comparison of performance with literature reported LIG/GO sensors

There are a few differences between our sensors and the sensors that are used by Zhu et al. [5], which can be divided into two main categories depending on their effect on the capacity response (and sensitivity). In the first category are included the parameters related to the fabrication process and the analysis of measurements with a minor effect on capacitance response, such as (i) the laser, (ii) the shape, and (iii) the range of the measurement. All of them can affect the sensitivity, however, their effect does not justify the 5 times difference (1800 vs 9150 pF/% RH). The laser can change the porosity of the electrodes, thus affecting the response time more than the sensitivity. On the other hand, the shape of the electrodes in combination with the laser can cause changes in the resistance of the electrodes. However, these modifications do not justify the recorded difference. The main effect of the shape regards the total length of the electrodes and the gap size, which are analysed below. As for the range of the measurement, our range is from 0 up to 97% RH while Zhu et al. start from 11% up to 97% RH, our sensitivity in the same range can be recalculated at 2030 pF/% RH.

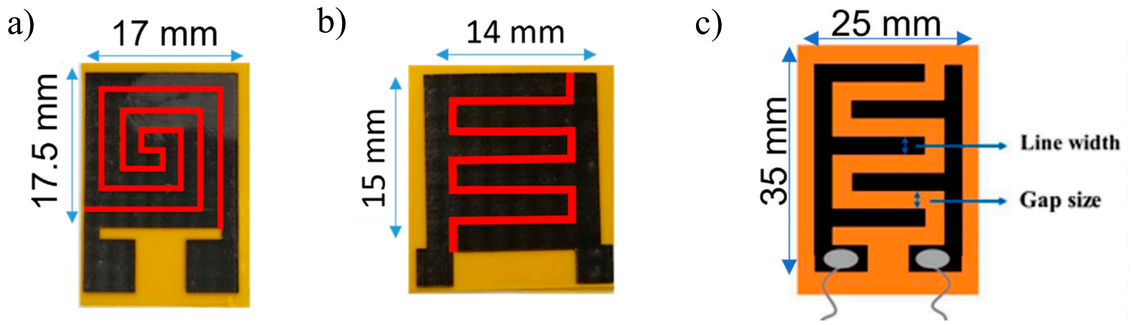

Figure S7, (a) and (b) the LIG sensors in our work and (c) general schematic representation of the sensors used by Zhu et al. [5]

The second category includes differences with a major effect on the capacitance response that can cause the sensitivity to reach high values (up to 5 times or even more). The differences are focused on (i) the total sensing area, (ii) the area of the plates of the IDE capacitors, (iii) the amount of GO, and (iv) the temperature of the measurements. Figure S7 presents the sensors used in both works, while Table S4 summarizes all their geometric characteristics. In our case, the sensing area of spiral and interdigitated sensors (Figure S7 a & b) are very close, 300 mm^2 and 210 mm^2 respectively, while in the case of Zhu et al., it is estimated to be between 875 mm^2 (25×35 mm^2) and 600 mm^2 (20×30 mm^2), assuming a frame of 2.5 mm around the electrodes. Thus, the difference in the sensing area of the sensors is more than 2 times (600/300), approaching even 4.1 times (875/210). On the other hand, the difference in the areas of the plates of the capacitors (red line on Figure S7 a & b) is greater, as summarized in Table S4. Assuming that the thickness of the LIG electrodes in all cases is equal, we can compare the plate areas of the capacitors by comparing their total length. In our case, the spiral sensor has a length of 111 mm, and the interdigitated 71 mm. In the paper of Zhu et al., the three different sensors were fabricated by changing the line width of the electrodes to 2, 1, and 0.2 mm, while the total area of the sensor (sensing area) and the gap size (0.2 mm) were kept constant.

By analysing the characteristic of those sensors, we can make a hypothesis about their total lengths. First of all, we can calculate the number of "*Lines*" by the following ratio:

$$N\#\ of\ Lines = \frac{Sensor\ length + Gap\ size}{Line\ width\ + Gap\ size}$$

Then, the total length is given by the equation:

$$Total\ length = (N\#\ of\ Lines - 1) \times [Sensor\ width - 2 \times (Line\ width\ + Gap\ size)] + Sensor\ length$$

As shown in Table S4, the total length of the electrodes of the sensors fabricated with 2, 1, and 0.2 mm line width was 228, 455, and 1460 mm, respectively. Comparing their capacitance performance using Fig. 4a on Zhu et al. paper, where it was used the same amount of GO for all the sensors, we can see that the capacitance at 97% RH increased as the total length of the electrodes increased. Namely, the capacitance of the sensor fabricated with a line width of 0.2 mm was ~1.8×10^6 pF, and it was ~4.5 times higher compared to the 1 mm line width sensor (~4×10^5 pF), and ~6.5 times higher compared to the 2 mm line width sensor (~2.7×10^5 pF), respectively, which are close to the ratio of the total length of their electrodes.

Table S4. Summary of the geometric characteristics for all the sensors in both publications.

Sensor	Sensor length (mm)	Sensor width (mm)	Line width (mm)	Gap size (mm)	N# of lines	Sensing area (mm^2)	Total length (mm)
Spiral (this work)	17.5	17	1.6	0.5	2	300	111
Interdigitated (this work)	15	14	2	0.2	6	210	71
Zhu et al. 0.1 mm	30	20	0.1	0.2	76	600	1460
Zhu et al. 1 mm	30	20	1	0.2	25	600	455
Zhu et al. 2 mm	30	20	2	0.2	14	600	228

Therefore, considering these geometric parameters, a comparison with our sensors may show that the lower capacitance response, and therefore the sensitivity, are partly related to the reduction of the total length of the electrodes, which are 13.1 and 20.5 times lower for the spiral and interdigitated sensors compared to the 0.2 mm line width sensor, respectively. In addition, the gap size in the case of Zhu sensors was ~300 μm lower compared to the spiral sensor, and as we know, the ratio of the plate's area to the distance of the plates (A/d) is proportional to the capacitance.

Another factor that enhances this behaviour is the amount of GO that was used for the fabrication of the sensors. In our case, the sensor with the optimum sensing properties was prepared by using 150 μl of GO from a 0.33 mg/ml dispersion, thus the mass of GO was 50 μg. On the other hand, the 0.2 mm line width sensor with the optimum sensing properties was fabricated by adding 0.1 ml of GO from a 2 mg/ml dispersion, therefore the mass of GO was four-times higher, at 200 μg. As it was shown from both works, the increase in the amount of GO increases the capacitance response, because more water molecules can be physiosorbed from the enhanced number of the oxygen functional groups. Here it should also be noted that in the work of Lan *et al.* [6] the amount

of GO was also higher, at 500 μg, while the sensitivity at the same frequency was more than 2 times lower. Table S5 presents a comparison based on the amount of GO used for the fabrication of the sensors in both works with their capacitance response at 97% RH and sensitivity in the range of 11-97% RH.

Table S5. Comparison of the amount of GO, capacitance response @ 97% RH, and sensitivity @ 11-97%.

Sensor	Spiral GO				0.2 mm line width		
	0.33 mg/ml	0.67 mg/ml	1.33 mg/ml	2 mg/ml	0.1 ml	0.5 ml	1 ml
Length/Gap	222				7300		
Sensing area (mm^2)	300				600		
GO mass (μg)	50	100	200	300	200	1000	2000
C (nF) @ 97%	175	345	520	1305	800	1800	2100
S (pF/% RH) @ 11-97% RH	2030	4010	6050	15180	9150	20930	24420

Most of the above parameters are related to the characteristics of the sensors, showing a lot of differences between the sensors of both works. In our case, we used 4 times lower amount of GO, in >2 times smaller sensing area, and 32 times smaller Length/Gap ratio. Despite those, our sensor achieves only 4.5 times lower capacitance response and sensitivity at the same range of RH, but at a lower temperature. At the same time, our sensor showed 3 times shorter response time and a very fast recovery time.

Table S6. Absolute humidity values at 22, and 27 °C for the tested RH levels

RH (%)	AH (g/m^3) @ 22 °C	AH (g/m^3) @ 27 °C
0	0	0
7.5	1.45	1.93
11	2.13	2.83
23	4.46	5.91
33	6.39	8.48
43	8.33	11.05
54	10.27	13.87
67	12.98	17.21
75	14.53	19.27
84	16.27	21.58
97	18.79	24.92

However, as was mentioned in the text, the capacitance response and therefore sensitivity can be affected also by other factors, which are not related to the sensor as a device, such as the resonant frequency of the measurements and the temperature. In both publications, the resonant frequency was the same (500 Hz). On the other hand, the temperature of the measurements in our case was

at 22 °C, and in Zhu et al. work 27 °C. The difference of 5 °C seems to be small, however, from the humidity point of view is not. As it was analysed in the text, the GO is absorbing water molecules from the atmosphere because of its oxygen functional groups, thus changing the capacitance of the sensor. The more water molecules in the atmosphere the more they will be adsorbed from the GO and subsequently cause an exponential increase in the capacitance. Thus, at 22 °C at 97% RH, there is 18.79 gr of water per m^3 of air, which is increased to 24.92 gr/m^3 at 27 °C [7-9]. Table S6 presents the absolute humidity values on all the tested relative humidity levels on both temperatures and is calculated based on the Magnus formula [10].

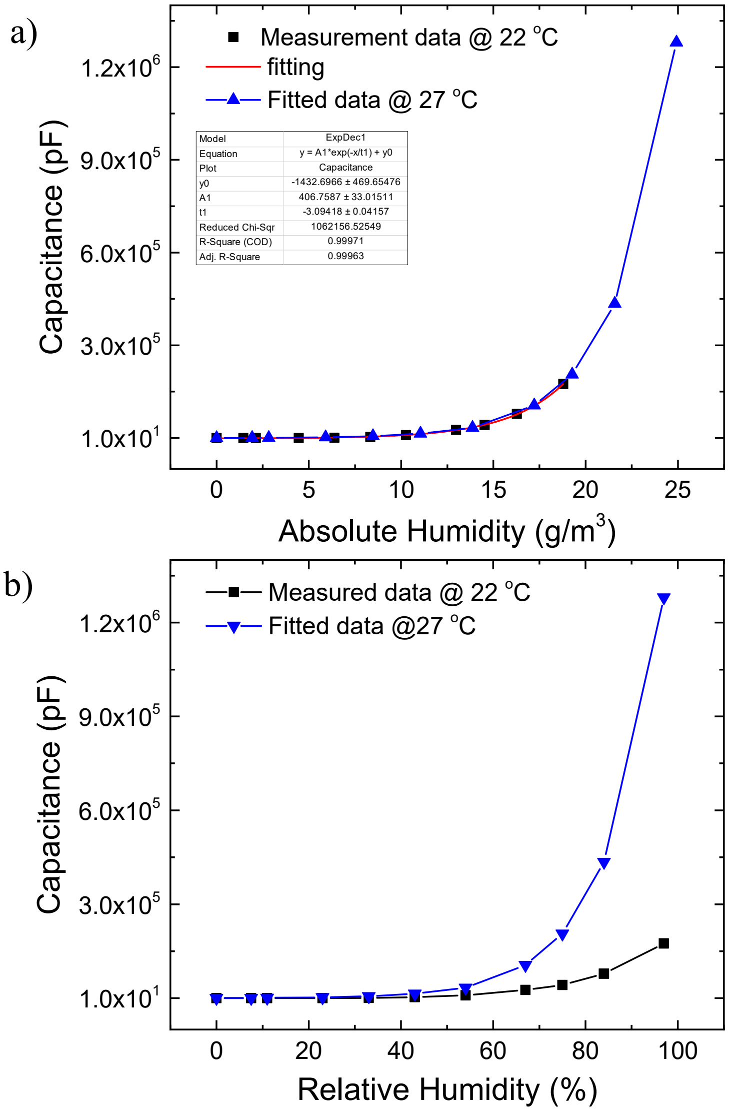

Figure S8. Capacitance response at 22 °C and the fitted values at 27 °C versus (a) Absolute humidity and (b) Relative humidity

The value of capacitance at 22 °C can be used in order to calculate the response of the sensor at 27 °C. Figure S8a presents the fitting of the capacitance values versus absolute humidity at 22 °C, while Figure S8b shows the capacitance response on all the tested RH levels for both temperatures.

The capacitance response of the sensor reaches the value of 1280000 pF at 97% RH at 27 °C, which corresponds to a sensitivity of 13190 pf/% RH in the range of 0-97% RH or 14870 pf/% RH in the range of 11-97% RH. Here, we must also note that the same fitting was used for the calculation of RH in Figure 6 on the text, where it was performed the comparison of our sensor with the commercial in one cooling and heating cycle.

References

1. Sygellou, L., et al., *Work Function Tuning of Reduced Graphene Oxide Thin Films.* The Journal of Physical Chemistry C, 2016. **120**(1): p. 281-290.
2. Ferrari, A.C., et al., *Raman Spectrum of Graphene and Graphene Layers.* Physical Review Letters, 2006. **97**(18): p. 187401.
3. Das, A., B. Chakraborty, and A.K. Sood, *Raman spectroscopy of graphene on different substrates and influence of defects.* Bulletin of Materials Science, 2008. **31**(3): p. 579-584.
4. Ferrari, A.C. and J. Robertson, *Interpretation of Raman spectra of disordered and amorphous carbon.* Physical review B, 2000. **61**(20): p. 14095.
5. Zhu, C., et al., *Graphene oxide humidity sensor with laser-induced graphene porous electrodes.* Sensors and Actuators B: Chemical, 2020. **325**: p. 128790.
6. Lan, L., et al., *One-step and large-scale fabrication of flexible and wearable humidity sensor based on laser-induced graphene for real-time tracking of plant transpiration at bio-interface.* Biosensors and Bioelectronics, 2020. **165**: p. 112360.
7. Buck, A.L., *New Equations for Computing Vapor Pressure and Enhancement Factor.* Journal of Applied Meteorology and Climatology, 1981. **20**(12): p. 1527-1532.
8. Seinfeld, J. and S. Pandis, *Atmospheric Chemistry and Physics. 1997.* New York, 2008.
9. Davis, R.E., G.R. McGregor, and K.B. Enfield, *Humidity: A review and primer on atmospheric moisture and human health.* Environmental Research, 2016. **144**: p. 106-116.
10. Alduchov, O.A. and R.E. Eskridge, *Improved Magnus form approximation of saturation vapor pressure.* Journal of Applied Meteorology and Climatology, 1996. **35**(4): p. 601-609.
